# The Influence of Social Determinants of Health on the Provision of Postpartum Contraceptives in Medicaid

**DOI:** 10.3390/healthcare10020298

**Published:** 2022-02-03

**Authors:** Irene Nsiah, Nidhi Vij Mali, Marie Barnard, Swarnali Goswami, Christy Lyle, Sujith Ramachandran

**Affiliations:** 1 Department of Pharmacy Administration, University of Mississippi, Oxford, MS 38677, USA; ibnsiah@go.olemiss.edu (I.N.); mbarnard@olemiss.edu (M.B.); sgoswami@go.olemiss.edu (S.G.); 2 Department of Public Policy Leadership, University of Mississippi, Oxford, MS 38677, USA; nvij@olemiss.edu; 3 Gainwell Technologies, 381 Highland Colony Parkway, Ridgeland, MS 39157, USA; christy.lyle@dxc.com; 4 Center for Pharmaceutical Marketing & Management, University of Mississippi School of Pharmacy, Oxford, MS 38677, USA

**Keywords:** maternal health, postpartum contraception, SDOH, LARC

## Abstract

Disparities continue to exist in the timely provision of postpartum contraception. This study aimed to identify prevalence and factors associated with postpartum contraception provision among women enrolled in Medicaid. A retrospective cohort study was conducted using the 2014 National Medicaid data, linked to county-level social vulnerability index (SVI) data. Women aged 15–44 with a live birth in 2014 were included. Multivariable logistic regression was used to predict 3-day provision of long-acting reversible contraception (LARC) and 60-day provision of most effective or moderately effective contraceptives (MMEC). Overall, 3-day LARC provision was 0.2% while 60-day MMEC was 36.3%. Significantly lower odds of receiving MMEC was found among women aged 15–20 (adjusted odds ratio [aOR] = 0.87; 95% CI:0.86–0.89) compared to women 20–44 years as well as among Asian women (aOR = 0.69; 95% CI:0.66–0.72) and Hispanic women (aOR = 0.73; 95% CI:0.72–0.75) compared to White women. The provision of postpartum contraception remains low, generally, and needs attention in communities experiencing poor maternal outcomes.

## 1. Introduction

The United States (US) is one of the few developed countries with an increase in maternal mortality rate (MMR) since 1990, with MMR increasing from 8.0 deaths per 100,000 in 1990 to 20.1 deaths per 100,000 live births in 2019 [1,2]. Pregnancy-related deaths occur most frequently during the postpartum period, and many are preventable [3,4]. Maternal mortality is often considered the ‘tip of the iceberg,’ with many additional women experiencing serious complications that have lifelong impact [5,6,7]. Severe maternal morbidity has a variety of negative outcomes, from increased rates of receiving a blood transfusion, hysterectomy, or ventilation to increased lengths of hospital stays and higher use of health services [8,9].

Within this context, postpartum contraception use can play a critical role in increasing the interpregnancy interval (IPI), which is essential for avoiding adverse maternal outcomes. Previous research has shown that short IPIs were associated with an increase in risk of adverse outcomes such as preeclampsia, uterine rupture, preterm birth, low-birth weight, and maternal death [10,11,12,13,14]. The American College of Obstetrics and Gynecology (ACOG) recommends that women should wait at least 6 months and up to 18 months between a live birth and the next conception in order to decrease the risk of poor maternal outcomes [15].

The provision of postpartum contraception immediately after delivery, and prior to discharge, has been shown to be most effective in reducing unintended pregnancies, and the ACOG recommends using contraceptives immediately after birth to increase the duration of the IPI [15]. Compared to individuals who receive postpartum contraception six to eight weeks after delivery, immediate intrauterine device (IUD) insertion can prevent an additional 88 unintended pregnancies over 2 years per 1000 women [16]. In addition, immediate provision of postpartum intrauterine contraception has been shown to be cost effective from the state’s perspective, with an estimated cost savings of $2.94 for every $1 spent on a state-financed IUD program [17]. 

The use of effective postpartum contraception methods remains low in the US, with a recent study showing that only about 53% of women reported using more effective methods (such as implants, IUDs, pills, rings and patches) and only 1 in 5 women report using most effective contraception such as implants and IUDs [18,19]. This is surprising as most women report an intention to use postpartum contraception [20]. In addition to barriers such as low income, low health literacy, and poor education, the Medicaid-eligible population needs special attention for the use of postpartum contraception because rates of unintended pregnancy in this population are significantly higher. Factors such as income, education, health literacy, and other socioeconomic factors are often referred to as social determinants of health (SDoH) and have been shown in previous research to be closely related to key preventive health behaviors [21]. 

It is important to understand the uptake of postpartum contraception, particularly in a Medicaid population that is vulnerable to several SDoH. Investigation of the factors associated with uptake can also help develop effective interventions to encourage use of postpartum contraception, taking into account maternal needs and wishes. The objectives of this study were (1) to evaluate the overall prevalence and patterns of state-level provision of postpartum contraception in Medicaid and (2) to assess the impact of SDoH on the rate of provision of postpartum contraceptives among women enrolled in Medicaid. 

## 2. Materials and Methods

A retrospective cohort study design was used to evaluate the patterns of state-level provision of contraceptives and the impact of SDoH on the provision of postpartum contraceptives among women in Medicaid in 2014. The study protocol was approved by the Institutional Review Board (IRB) at the University of Mississippi, and the use of Medicaid data were covered under a data use agreement with the Centers for Medicare and Medicaid Services (DUA# RSCH-2017-51606).

### 2.1. Data Sources

This study was conducted using the Medicaid administrative claims data from 17 states (CA, GA, ID, IA, LA, MI, MN, MS, MO, NJ, PA, SD, TN, UT, VT, WV, WY) for the year 2014. Medicaid is a public entitlement program jointly funded by the federal government and states to provide health insurance to individuals with low income. Each state operates its own Medicaid program under broad guidance from the federal government, which gives states considerable flexibility in how they design and administer the program. However, federal law requires states to provide certain mandatory benefits, including inpatient and outpatient hospital services, physician services, home health services, nursing facility services, etc. As of June 2021, 83.2 million Americans were enrolled in Medicaid, including eligible low-income adults, pregnant women, children, elderly adults and individuals with disabilities [22]. Medicaid administrative claims data contain de-identified information pertaining to over 25 million Medicaid beneficiaries. The data contain an inpatient claims database, an outpatient claims database, a pharmacy claims database, and a beneficiary master file that provides information about demographics, eligibility, and zip code of residence. To identify SDoH factors, each beneficiary’s zip code was used to determine the county of residence and linked to county-level Social Vulnerability Index (SVI) data from the Centers for Disease Control and Prevention (CDC) for the year 2014 [23]. Previous studies have demonstrated that the SVI is a robust predictor of several health indicators such as poor outcomes after surgical episodes [24,25], preventative health behaviors such as physical activity [26,27] and vaccination rates [28], and even outcomes from the COVID-19 pandemic [29,30].

### 2.2. Study Population

The study population was identified based on the Office of Population Affairs (OPA) Contraceptive Care–Postpartum Women Ages 15–44 quality measure [31]. Per the OPA Contraceptive Care–Postpartum (CCP) measure, individuals were included if they were aged 15–44 years as of 31 December 2014, with a live birth in 2014 and were continuously enrolled in Medicaid for at least 60 days from the date of delivery. The date of live delivery was defined as the index date and identified using ICD-9 diagnoses and procedure codes provided by OPA as part of the CCP measure. Deliveries that did not end in a live birth and deliveries that occurred during the last two months of 2014 were excluded. For the second objective, additional exclusion criteria were applied. Individuals who had multiple deliveries, as well as deliveries occurring after 30 September 2014, were excluded so as to capture continued eligibility beyond 60 days postpartum. 

### 2.3. Postpartum Contraception Provision

The provision of contraceptives was identified using the OPA CCP measure [31]. This included most or moderately effective (MMEC) FDA-approved methods of contraception and long-acting reversible methods of contraception (LARC) within 60 days of delivery. The most effective methods of contraceptives included female sterilization, contraceptive implants, or intrauterine devices or systems (IUD/IUS). Moderately effective contraceptives included the use of injectables, oral pills, patch, ring, or diaphragm, and LARCs included the use of contraceptive implants and IUD/IUS. The use of contraceptives was identified using national drug codes (NDCs) provided by OPA as part of the CCP measure. 

### 2.4. Theoretical Framework

**Figure 1:**
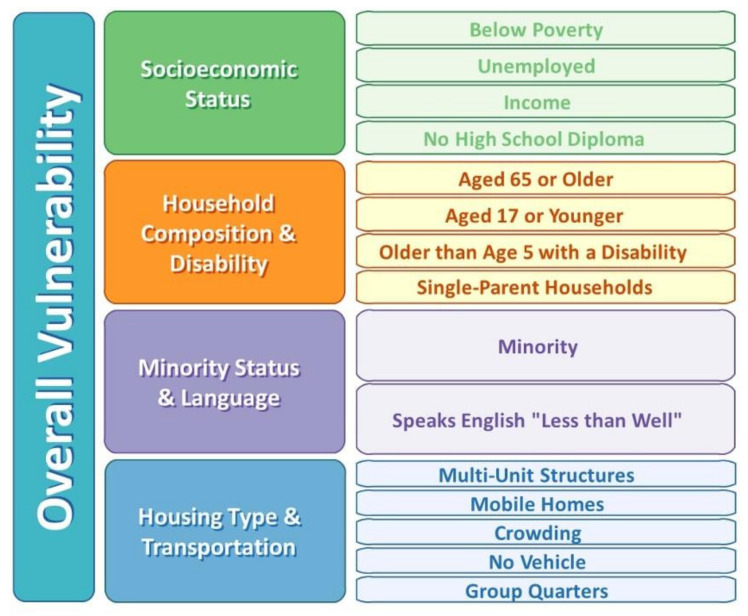
CDC SVI variables and themes. Description: The CDC SVI is derived from 15 social factors and arranged in 4 themes. The SVI is a measure of the relative vulnerability of every US Census tract to external stresses. Abbreviations: CDC—Centers for Disease Control and Prevention; SVI—Social Vulnerability Index. Data source: Centers for Disease Control and Prevention (CDC). CDC/ATSDR Social Vulnerability Index (SVI). Available online: https://www.atsdr.cdc.gov/placeandhealth/svi/documentation/SVI_documentation_2014.html (accessed on 20 December 2021).

In order to estimate the impact of SDoH on CCP, the Healthy People 2020 Social Determinants of Health framework was utilized, as applied by the CDC SVI [23]. The Healthy People 2020 framework emphasizes the collective impact and influence of determinants such as physical and social environment, individual behavior, health services and biology and genetics on the health outcomes of a population [32]. The CDC SVI employs this framework to assess the relative vulnerability of every US Census tract or county to external stresses such as natural or human-caused disasters or disease outbreak. The higher the ranking, the more vulnerable the geographic region. The SVI ranking is derived from 15 social factors, based on variables captured by the American Community Survey (ACS) data, arranged into four themes: socioeconomic status, household composition and disability, minority status and language, and housing type and transportation. Additional information on the variables used and the four themes are provided in Figure 1. Data from the SVI were classified according to whether a county was ranked in the top quartile, the bottom quartile, or the middle for each of the four themes before they were linked to patient records.

In addition to the SVI, in an effort to assess the impact of state Medicaid policies around the continued provision of Medicaid eligibility during the postpartum period, beneficiaries were flagged if they had more than 60 days of continuous enrollment beyond the delivery date. Additionally, the models predicting the use of postpartum contraceptives also adjusted for demographic factors such as age and race of the individual. Age and race were obtained from the Medicare beneficiary master file. Similar to how the CCP measure is used, beneficiaries age was classified as 15–20 and 21–44. Race/ethnicity categories included White, Black, Hispanic, Asian and Other/Unknown race. 

### 2.5. Statistical Analysis

Baseline descriptive statistics were estimated and the prevalence of postpartum contraceptive use (MMEC/LARC) was also estimated in each available state to facilitate comparisons to a national rate. Multivariable logistic regression models were used to assess the relationship between SDoH factors and the provision of LARC and MMEC during the 3- and 60-day postpartum periods, respectively, and to estimate adjusted odds ratios (aOR) and 95% confidence intervals (CI). All analyses were conducted using SAS 9.4 (SAS Institute, Cary, NC, USA), and an a priori significance level of *p* < 0.05 was used for all analyses. 

## 3. Results

**Figure 2:**
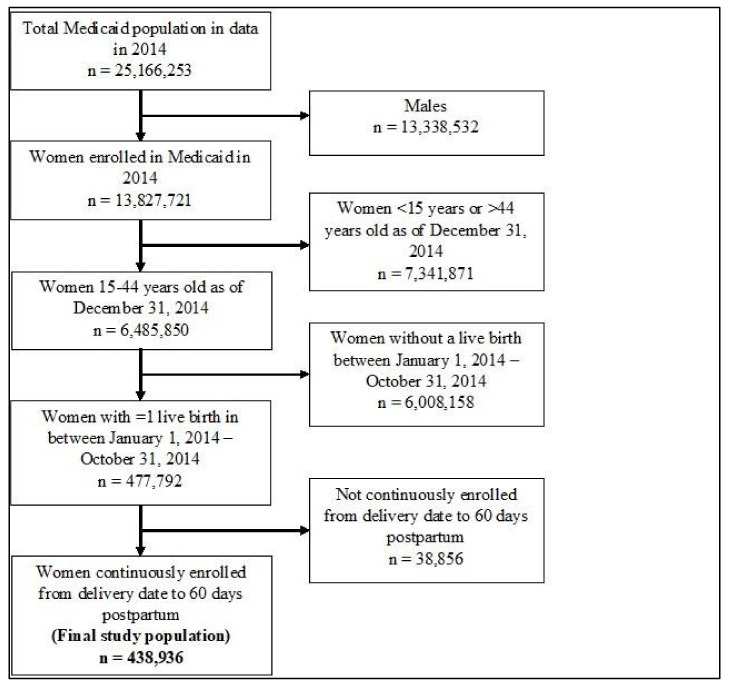
Sample selection of eligible population in Medicaid 2014. Description: Inclusion and exclusion criteria were applied to the Medicaid population to identify individuals who qualified for final inclusion in the study.

A total of 438,936 women were included in the study after applying all inclusion and exclusion criteria (Figure 2), with a majority of study participants aged 21–44 years (85.9%). The composition of the study participants by race was as follows: 44.0% White women, 24.9% Black women, 2.7% Asian women, 19.0% Hispanic women, and 9.3% other/unknown race (Table 1).

Table 2 shows the provision of postpartum contraception. The overall provision of LARC during the 3-day postpartum period was 0.2%, while the overall rate for MMEC during the 60-day postpartum period was 36.3%. In general, MMEC rates during the 60-day postpartum period were slightly higher in women aged 21–44 years (36.6%) compared to women aged 15–20 years (34.3%, *p* < 0.001), in White women (40.5%) compared to Black women (38.4%), Asian women (27.0%) or Hispanic women (28.3%) (*p* < 0.001). 

There was significant variation in MMEC across the 17 states included in the study (Table 2). The top three states with the highest rates for MMEC during the 60-day postpartum period were Mississippi (MS) (48.5%), Louisiana (LA) (48.2%) and Tennessee (TN) (48.1%). A majority of the other states had rates between 37% and 45%. While rates for California (CA) (24.8%) and New Jersey (NJ) (30.5%) were lower compared to the national rate of 36.3%, the measure rate for West Virginia (WV) was much lower, at 7.1%. For LARC provision during the 3-day postpartum period, the states with the highest rates included Iowa (IA) (0.7%), Pennsylvania (PA) (0.5%) and Vermont (VT) (0.5%).

The results of the multivariable logistic regression models predicting the receipt of MMEC and LARC among Medicaid eligible women are provided in Table 3. In general, women aged 15–20 years had lower odds of receiving MMEC during the 60-day postpartum period (aOR = 0.87; 95% CI: 0.86, 0.89; *p* < 0.001) but higher odds of receiving LARC during the 3-day postpartum period (aOR = 1.37; 95% CI: 1.14, 1.64; *p* = 0.001) compared to women aged 21–44 years. Compared to White women, Asian women (aOR = 0.69; 95% CI: 0.66, 0.72; *p* < 0.001) and Hispanic women (aOR = 0.73; 95% CI: 0.72, 0.75; *p* < 0.001) had lower odds of receiving MMEC. There was no significant difference in the odds of MMEC receipt for Black women (aOR = 1.00; 95% CI: 0.98, 1.01; *p* = 0.753) compared to White women. Black women (aOR = 2.91; 95% CI: 2.43, 3.49; *p* < 0.001) had significantly higher odds of receiving LARC during the 3-day postpartum compared to White women. There was no significant difference between Asian women (aOR = 1.19; 95% CI: 0.74, 1.91; *p* = 0.486) and White women, while Hispanic women (aOR = 0.76; 95% CI: 0.58, 0.99; *p* = 0.039) had lower odds of receiving LARC compared to White women. Although women who were enrolled in Medicaid beyond the 60-day postpartum period had lower odds of receiving MMEC (aOR = 0.97; 95% CI: 0.95, 0.98; *p* < 0.001), they had higher odds of receiving LARC (aOR = 1.32; 95% CI: 1.11, 1.58; *p* = 0.002) compared to women whose enrollment was discontinued at the end of the 60-day postpartum period.

In terms of SDoH factors, measured using the SVI themes, living in the most vulnerable counties (top quartile) under the socioeconomic theme (aOR 1.07; 95% CI: 1.04, 1.09; *p* < 0.001) and household composition and disability (aOR = 1.37; 95% CI: 1.34, 1.41; *p* < 0.001) were associated with higher odds of MMEC provision compared to living in the least vulnerable counties (bottom quartile). Conversely, living in the most vulnerable quartile in terms of minority status and language (aOR = 0.86; 95% CI: 0.84, 0.89; *p* < 0.001) and housing and transportation (aOR = 0.92; 95% CI: 0.90,0.94; *p* < 0.001) were associated with lower odds of MMEC provision during the 60-day postpartum period. Compared to living in least vulnerable counties, living in the most vulnerable counties in terms of socioeconomic theme (aOR = 0.76; 95% CI: 0.58, 0.99; *p* = 0.043), household composition and disability (aOR = 0.27; 95% CI: 0.20, 0.38; *p* < 0.001) were associated with lower odds of LARC provision, while housing and transportation (aOR = 2.44; 95% CI: 1.72, 3.45; *p* < 0.001) was associated with significantly higher odds of LARC provision.

## 4. Discussion

The results of this study add to the existing evidence around the provision of timely postpartum contraception in the US. LARC provision during the 3-day postpartum period was 0.2%, while only about one in three women received MMEC during the 60-day postpartum. In addition, the provision of postpartum contraception was closely tied to sociodemographic and SDoH factors, and significant variation in postpartum contraception provision was observed across states.

The MMEC rates from this study using data from 2014 are slightly lower than more recent estimates from the Medicaid Adult Core Set, which reported an overall rate of 40.4% in 2020 [33]. In addition, while LARC rates have improved from 2014, at 2.2% in 2020, rates are still low [33]. These rates are surprising for several reasons. First, there have been various initiatives at the federal level aimed at improving access to postpartum contraception. In 2014, the Center for Medicaid and CHIP Services (CMCS) launched the Maternal and Infant Health Initiative in partnership with states and Medicaid providers, with the goal of increasing the use of MMEC among women in Medicaid and CHIP, especially during the postpartum period [34]. In addition, one of the Healthy People 2020 goals was to increase the proportion of women delivering a live birth who used an MMEC by 10% [35]. There have also been several state-level initiatives, with most states publishing guidance around reimbursement for immediate LARC insertion [36]. 

However, the 2020 rates from the Medicaid Adult Core Set show that there has been little improvement in use of postpartum contraception from the rates found in the current study. This is corroborated by a recent study which found a very small increase of only about two percentage points in LARC uptake after Ohio’s Medicaid expansion [37]. The lack of improvement in postpartum contraception uptake may be due to a lack of knowledge about availability and recent policy changes that have significantly reduced the cost of postpartum contraception. Low rates of contraceptive use may also be due to hesitancy on the part of providers to avoid the impression of being paternalistic or coercive in their attitude towards recommending postpartum contraception. While it is important that the use of postpartum contraception be the woman’s choice, it is equally important that there is a conversation about the topic. Pregnant women should be made aware of these options during prenatal counseling, and although it should not be forced on them, they should be able to understand the importance of postpartum contraception use and make an informed decision based on the options available to them and their own reproductive needs. 

This study found interesting variations in sociodemographic, SDoH, and geographic rates of postpartum contraception provision. In general, women aged 15–20 years had higher odds of receiving LARC. These results have been corroborated by other studies, which have found a positive association between younger age and use of more effective postpartum contraception [38]. Conversely, the finding from this study that Black women had significantly higher odds of receiving LARC during the 3-day postpartum period compared to White women is different from a study by Thiel de Bocanegra et al., which found that Black women had lower odds of receiving LARC compared with White women [39]. The differences observed may be due to several reasons. First, the Thiel de Bocanegra et al. study focused on California Medicaid, while this study included data from 17 states. In addition, LARC provision for this study was measured over a 3-day period, while the Thiel de Bocanegra et al. study examined LARC over a 99-day period. 

Of great interest is the finding from this study that a woman’s residing in the most vulnerable counties was significantly associated with provision of both LARC and MMEC. However, variable associations were observed between CDC’s themes of social vulnerability, LARC provision and MMEC uptake. For example, women residing in the most vulnerable counties in terms of housing and transportation had greater odds of LARC provision, whereas those living in counties with vulnerable socioeconomic status (which includes living below poverty, unemployment, low income, no high school diploma) had fewer odds of LARC provision. Similarly, women living in the most vulnerable counties in terms of socioeconomic status, household composition and disability status (age 65 years or older or 17 years or younger, older than age 5 with a disability, or single parent households) had higher odds of MMEC uptake in the 60-day postpartum period, whereas those residing in the most vulnerable counties in terms of minority status and language as well as housing and transportation had lower odds of MMEC uptake. To our knowledge, this is the first study to examine postpartum contraception provision in the context of social vulnerability. The variable findings in this study are likely explained by a complex interplay of the various social determinants that drive healthcare decisions and outcomes. For example, it is possible that residing in locations with transportation issues may lead individuals to choose immediate LARC insertion after delivery so as to avoid traveling to a clinic for follow up appointments. Vulnerability in terms of household composition, which may be related to greater caregiving needs or lack of availability of childcare, may in turn motivate uptake of postpartum contraceptives. In addition, among Medicaid beneficiaries, the ability to pay for contraceptive medications continues to be a significant challenge. This is particularly relevant in 2014, this study’ time period, because it was prior to when most states provided reimbursement for immediate LARC insertion. While further research is needed to explore the interplay of these indicators of vulnerability, individual race or ethnicity, and postpartum contraceptive use, it is clear that women residing in counties with greater vulnerability need immediate attention from healthcare providers and policymakers alike in order to improve the quality of maternal care and outcomes from pregnancy.

### 4.1. Implications for Policy and/or Practice

While this study used data from 2014 to estimate use of postpartum contraception, the relationships found here may still be valuable. For instance, the American College of Obstetricians and Gynecologists (ACOG) recommends immediate postpartum LARC insertion (within 10 min after delivery) [40], and the low rates of post-partum contraceptive use both in this study and that found in recent literature [33] suggest that providers and patients may not be adhering to the ACOG recommendations. This study highlights the importance of counseling in addition to access. It is not sufficient that reimbursement for services is available; patients should be made aware of these services. While this study did not evaluate receipt of such services, clinicians should endeavor to incorporate prenatal counseling around postpartum contraception use as part of routine prenatal care. In addition, given the significant variation observed across states in postpartum contraception provision, states with lower rates should consider targeted interventions and policies to improve access to postpartum services, to ensure that women residing in vulnerable regions are aware of postpartum services and able to access these services as needed. 

### 4.2. Limitations

There are several limitations inherent in the use of administrative claims database. Administrative claims data do not capture services paid in cash, so any use of postpartum contraception, especially of moderate contraception that may have been obtained outside the hospital and paid in cash will not be captured, meaning actual use may be underestimated. In addition, due to limitations in administrative claims data, which does not collect information about SDoH factors, individual level factors could not be assessed, and therefore the analysis was conducted at the county level. As such, the results from this study are susceptible to ecological fallacy and inferences about individual behavior should be made with caution. Further, the data used in this study are from 2014 prior to policy changes that many states may have implemented with respect to reimbursement for maternal care, in general, or contraceptive use, specifically. Therefore, the rates presented in this study may not be representative of the rates of contraceptive use today. While several states had chosen to expand Medicaid in 2014, this study elected not to account for Medicaid expansion status because Medicaid enrollment was directly captured at the individual level. Nevertheless, there may be other differences between individuals living in expansion vs. non-expansion states that may not be fully captured by whether or not a state chose to expand Medicaid. Future research should examine the impact of Medicaid expansion on postpartum contraception over and above Medicaid enrollment. Finally, the results of this study are only representative of the Medicaid population beneficiaries from the 17 states used in the analysis and may not be generalizable to all states.

## 5. Conclusions

Immediate and timely provision of effective postpartum contraception have been shown to be very effective in reducing closely spaced pregnancies. However, the provision of effective postpartum contraception remains low in the US, with significant sociodemographic, SDoH, and geographic variations. Targeted interventions by federal and state healthcare providers and payers are needed to ensure that women are aware of the availability of postpartum contraception and are able to access it as needed.

## Figures and Tables

**Table 1:** Baseline Descriptives for Study Population (*n* = 438,936).

Characteristic	*n*	%
Age group		
15–20 years	61,751	14.07%
21–44 years	377,186	85.93%
Race/ethnicity		
White	193,594	44.11%
Black	109,319	24.91%
Asian	11,808	2.69%
Hispanic	83,490	19.02%
Other/Unknown	40,725	9.28%
State		
CA	129,625	29.53%
GA	50,115	11.42%
IA	11,610	2.65%
ID	7249	1.65%
LA	27,668	6.3%
MI	37,850	8.62%
MN	18,271	4.16%
MO	21,594	4.92%
MS	18,325	4.17%
NJ	19,276	4.39%
PA	42,972	9.79%
SD	3167	0.72%
TN	30,667	6.99%
UT	9426	2.15%
VT	2181	0.5%
WV	6787	1.55%
WY	2153	0.49%

**Table 2:** Prevalence of Provision of 3-Day LARC and 60-Day MMEC Postpartum Contraception.

Characteristic	3-Day LARC Provision Rate		*p*-Value	60-Day MMEC Provision Rate		*p*-Value
	*n*	%		*n*	%	
Overall	787	0.18%		160,615	36.31%	
Age group						
15–20 years	141	0.23%	0.002	21,343	34.29%	<0.001
21–44 years	646	0.17%		139,272	36.64%	
Race/ethnicity						
White	220	0.11%	<0.001	78,926	40.48%	<0.001
Black	382	0.35%		42,299	38.41%	
Asian	20	0.17%		3225	27.05%	
Hispanic	95	0.11%		23,780	28.25%	
Other/Unknown	70	0.17%		12,385	30.07%	
State						
CA	172	0.13%	<0.001	32,467	24.84%	<0.001
GA	29	0.06%		21,580	42.94%	
IA	85	0.73%		4685	40.15%	
ID	1	0.01%		3089	42.15%	
LA	59	0.21%		13,400	48.18%	
MI	15	0.04%		14,578	38.31%	
MN	49	0.27%		7058	38.34%	
MO	51	0.23%		9757	44.80%	
MS	8	0.04%		8982	48.52%	
NJ	8	0.04%		5910	30.54%	
PA	233	0.54%		16,075	37.13%	
SD	4	0.12%		1325	41.01%	
TN	43	0.14%		14,973	48.14%	
UT	18	0.19%		4262	44.21%	
VT	11	0.50%		1013	46.15%	
WV	0	0.00%		488	7.10%	
WY	1	0.05%		973	44.23%	

Abbreviation: LARC, long-acting reversible contraception; MMEC, most or moderately effective contraception.

**Table 3:** Multivariable Logistic Regression Predicting Provision of Postpartum Contraception Among Medicaid Eligible Women.

Characteristic	3-Day LARC Provision		60-Day MMEC Provision	
	aOR (95% CI)	*p*-Value	aOR (95% CI)	*p*-Value
Age Group (Ref = 21–44 years)				
15–20 years	1.37 (1.14, 1.64)	0.001	0.87 (0.86,0.89)	<0.001
Race/ethnicity (Ref = White)				
Black	2.91 (2.42, 3.49)	<0.001	1.00 (0.98,1.01)	0.756
Asian	1.19 (0.74, 1.91)	0.486	0.69 (0.66,0.72)	<0.001
Hispanic	0.76 (0.58, 0.99)	0.039	0.73 (0.72,0.75)	<0.001
Other/Unknown	1.29 (0.98, 1.70)	0.072	0.73 (0.72,0.75)	<0.001
Medicaid Eligibility >60 days postpartum (Ref = Discontinued)				
Continued	1.32 (1.11, 1.58)	0.002	0.97 (0.95,0.98)	<0.001
Social Vulnerability Index				
Socioeconomic Theme (Ref = Least vulnerable–Bottom Quartile)				
Moderately vulnerable (Q2/Q3)	0.41 (0.34, 0.51)	<0.001	1.00 (0.98,1.02)	0.932
Most vulnerable (Top Quartile)	0.76 (0.58, 0.99)	0.043	1.07 (1.04,1.09)	<0.001
Household Composition and Disability (Ref = Least vulnerable–Bottom Quartile)				
Moderately vulnerable (Q2/Q3)	0.81 (0.68, 0.96)	0.014	1.27 (1.25,1.29)	<0.001
Most vulnerable (Top Quartile)	0.27 (0.20, 0.38)	<0.001	1.37 (1.34,1.41)	<0.001
Minority status and Language (Ref = Least vulnerable–Bottom Quartile)				
Moderately vulnerable (Q2/Q3)	0.63 (0.45, 0.87)	0.005	1.18 (1.15,1.21)	<0.001
Most vulnerable (Top Quartile)	0.77 (0.55, 1.08)	0.126	0.86 (0.84,0.89)	<0.001
Housing and Transportation (Ref = Least vulnerable–Bottom Quartile)				
Moderately vulnerable (Q2/Q3)	2.01 (1.45, 2.78)	<0.001	0.98 (0.96,1.00)	0.070
Most vulnerable (Top Quartile)	2.44 (1.72, 3.45)	<0.001	0.92 (0.90,0.94)	<0.001

Abbreviation: LARC, long-acting reversible contraception; MMEC, most or moderately effective contraception; aOR, adjusted odds ratio; CI, confidence interval; Ref, reference group; Q2, Quartile 2; Q3, Quartile 3.

## Data Availability

Restrictions apply to the availability of these data. Data was obtained from the Centers for Medicare and Medicaid Services and governed by a Data Use Agreement. As such the data cannot be made publicly available.
